# Dielectric-Free Molybdenum Disulfide Transistors with In-Plane Gates

**DOI:** 10.1021/acsami.4c18855

**Published:** 2025-03-12

**Authors:** Che-Jia Chang, Shih-Jie Chen, Tzu-Hsuan Chang, Po-Tsung Lee, Shu-Wei Chang, Shih-Yen Lin

**Affiliations:** † Graduate Institute of Electronics Engineering, National Taiwan University, No. 1, Sec. 4, Roosevelt Road, Taipei 10617, Taiwan; ‡ Research Center for Applied Sciences, Academia Sinica, No. 128, Sec. 2, Academia Road, Taipei 11529, Taiwan; § Department of Photonics, National Yang Ming Chiao Tung University, No. 1001, Daxue Road, East District, Hsinchu 300, Taiwan

**Keywords:** in-plane gates transistors, 2D materials, interfaces, current saturation, film transferring

## Abstract

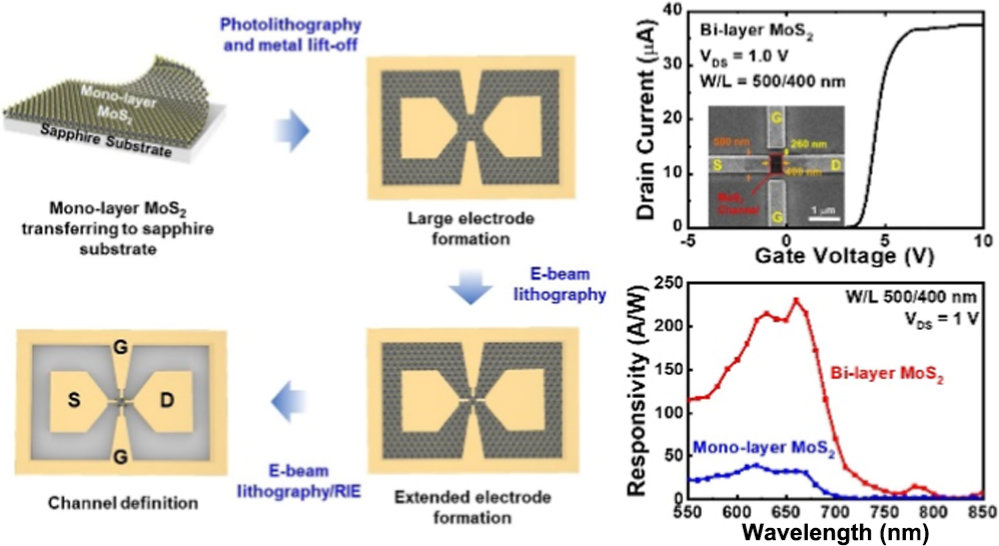

In this work, we realize bilayer molybdenum disulfide (MoS_2_) transistors with in-plane gates on sapphire substrates. Through sequential transferring of MoS_2_, e-beam lithography, and metal lift-off, a device with channel width/length of 500:400 nm is fabricated. With a 250 nm separation between the in-plane gate and MoS_2_ channel, a drain current as high as 37 μA with a clear saturation region is observed. The device shows a high ON/OFF current ratio over 10^9^, small gate bias required for current modulation, and high responsivity about 230 A/W when operated in the phototransistor mode. The gain of the phototransistor reaches 432. A quick calculation from the transfer curve using the formula of conventional transistors gives a field-effect mobility of 6365.9 cm^2^·V^–1^·s^–1^, which, although overestimated, still suggests good performance of the device. With an additional MoS_2_ layer to isolate the MoS_2_ channel from the influence of the substrate, this dielectric-free in-plane gate transistor has exhibited potential in electronics. The high responsivity of the device under relatively low applied voltages is also promising for weak-light detection.

## Introduction

Since the discovery of graphene in 2004, the possibility of implementing high-performance electronic devices with lateral current flows in a few atomic layers has made two-dimensional (2D) materials promising candidates for integrated circuits in the nanometer regime.^1^ Due to the thin-body nature of graphene, one of its major applications is aimed at electronic devices in the technology node below 1 nm. However, despite the high mobility, the absence of a bandgap in graphene results in no clear OFF state in graphene transistors. Perusing high ON/OFF ratios necessary for logic circuits, research groups around the world turn their attention to other 2D materials with useable bandgaps.^2−7^ Among all semiconductor 2D materials, molybdenum disulfide (MoS_2_) is the most discussed.^8−10^ Compared with other semiconductor 2D materials, it is relatively easy to fabricate wafer-scale MoS_2_ films using chemical vapor deposition (CVD).^11,12^ Nowadays, electrical and optical devices based on monolayer MoS_2_ have been widely reported in the literature.^2,8−10,13,14^ With the introduction of polycrystalline antimonene contact electrodes which show a low contact resistance with MoS_2_, high-performance transistors have been demonstrated.^15,16^ A high responsivity helpful for weak light detection is also reported in plasmonic-assisted MoS_2_ phototransistors.^17^ These extraordinary performances have revealed the advantages of 2D materials for nanoscale device applications.

While the thin body nature of 2D materials is advantageous for electronic device applications, the influence of interfaces, such as those between the channel and dielectric, can significantly degrade device performance.^18,19^ It has been shown that a monolayer MoS_2_ layer can shield the channel from the influence of dielectric layers. A significant improvement over device performance has been observed in trilayer MoS_2_ transistors with all 2D material interfaces.^20^ These findings highlight the importance of mitigating the impact of non-2D material interfaces for transistors based on the 2D material. Still, one protective layer of MoS_2_ may not completely remove the effects of dielectric layers. It is crucial to develop strategies to protect 2D material channels from those effects. One possible approach is the architecture of in-plane gate transistors (IPGTs).^21−23^ It is possible to achieve current modulations without gate dielectrics in these devices. In this paper, we fabricate MoS_2_ IPGTs with a gate-to-channel separation of 250 nm using e-beam lithography on sapphire substrates. In the presence of a protective layer MoS_2_ to isolate the influence of the sapphire substrate to the monolayer MoS_2_ channel, an ON/OFF ratio over 10^9^ is observed from the MoS_2_ IPGTs. Based on the formula of the conventional transistor, a high field-effect mobility of 6365.9 cm^2^·V^–1^·s^–1^ is also observed from the device. With the high photoconductivity due to short channel and photogating effect,^24^ the device exhibits an ultrahigh peak responsivity of 230 A/W at the wavelength of 660 nm at *V*_DS_ = 1.0 V. The results have opened up new possibilities for the practical applications of 2D materials based on the architecture of IPGT and multilayer MoS_2_ channels.

## Results and Discussion

**Figure 1:**
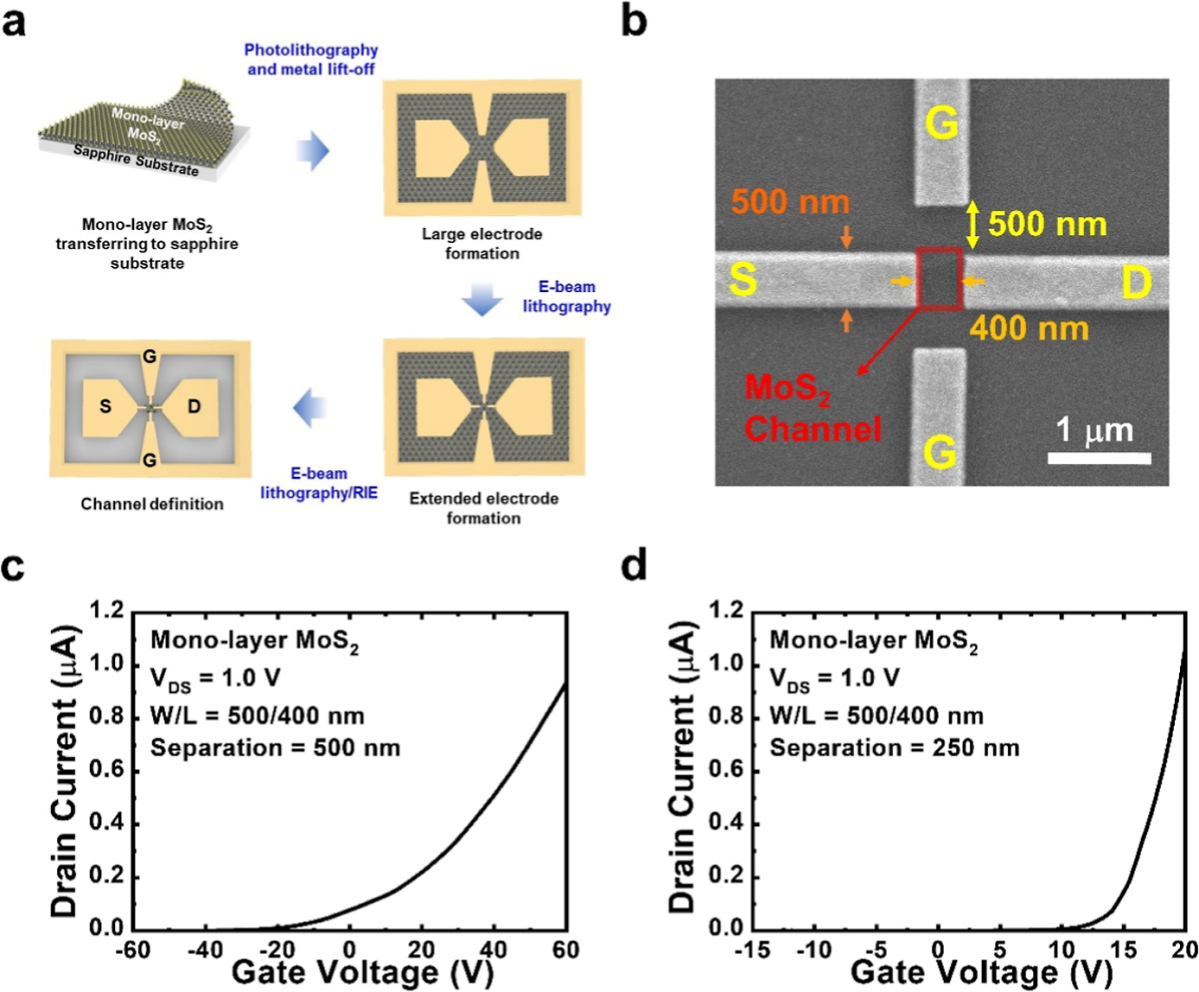
(a) Fabrication procedure of monolayer MoS_2_ IPGTs. (b) SEM image and (c) transfer curve at *V*_DS_ = 1.0 V of the device with 500 nm gate-to-channel separation. (d) Transfer curve at *V*_DS_ = 1.0 V of the device with 250 nm gate-to-channel separation.

The fabrication process of monolayer MoS_2_ IPGTs is shown in Figure 1a. To maintain the same condition of film attachment for each layer of MoS_2_ to the sapphire substrate, the as-grown monolayer MoS_2_ using CVD growth on a sapphire substrate is transferred to another sapphire substrate through the stamping of polydimethylsiloxane (PDMS). After the transfer of MoS_2_ film, large electrodes with 100 nm Au deposited at 75 °C are formed with photolithography and metal lift-off for device measurement. The deposition temperature 75 °C of the Au electrodes, which is slightly higher than room temperature, is to reduce the contact resistance at the Au/MoS_2_ interface and not to deteriorate the photoresist for the metal lift-off.^15,25^ Electron-beam lithography is then employed for the deposition of a much thinner extended Au electrode and definition of the channel. The channel is defined through reactive ion etching (RIE) with CF_4_ plasma. The scanning electron microscopy (SEM) image of the extended electrodes and MoS_2_ channel is shown in Figure 1b. The channel width/length of the device is 500:400 nm, and the gate-to-channel separation is 500 nm. The transfer curve of the device in Figure 1c shows clear drain current modulations, indicating that the IPGT device architecture can also be applied to 2D materials. However, a large gate voltage is still required to turn on and off the transistor, and the ON/OFF ratio is only about 1 × 10^4^. It is suggested that the 500 nm air gap between the in-plane gates and the MoS_2_ channel may be too large for efficient current modulations. The transfer curve in the logarithmic scale (Figure S1a in the Supporting Information) shows a relatively high off current for the device.

To further enhance the modulation efficiency, we reduced the gate-to-channel separation to 250 nm. The SEM image of the device is shown in Figure S2 of the Supporting Information, and the corresponding transfer curve is presented in Figure 1d. Compared with the device with 500 nm separation, the one with 250 nm separation requires only about 20 V gate voltage to reach the drain current level of 1 μA. Its ON/OFF ratio is also enhanced to 10^7^, reflecting that the in-plane gates with a smaller gate-to-channel separation of 250 nm can turn off the device more effectively. The transfer curve in the logarithmic scale (Figure S1b in the Supporting Information) shows a reduced off current for the device. Using the expression μ = (d*I*_DS_/d*V*_GS_) × (*L*/*W*) × (*t*/ε) × *V*_DS_^–1^, we calculate the field-effect mobility of the device, where the channel width *W* is 500 nm, the channel length *L* is 400 nm, the dielectric constant ε is ε_0_ (air), and the oxide thickness *t* is 250 nm (the separation between the gate and MoS_2_ channel). Such calculations of the field-effect mobility may cause deviations (often overestimations) because the electric-field lines between the gate and channel are distributed both in the air and substrate and are generally nonuniform, which in turns induces an uneven carrier profile in the channel (effectively leading to a larger ε and a wider *W*).^26,27^ In fact, the channel width *W* and dielectric constant ε in the formula should be replaced by effective ones parametrized by the details of the in-plane gate. A comprehensive calculation of the field-effect mobility of IPGTs will be conducted in the future. For a quick estimation of device performance, we used the above expression of field mobility. The field-effect mobility thus obtained is 67.9 cm^2^·V^–1^·s^–1^. Compared with the single-digit field-effect mobility (in cm^2^·V^–1^·s^–1^) of monolayer MoS_2_ bottom-gate (BG) transistors, the relatively high mobility here indicates that some phenomenon that favors carrier transport may take place in devices with reduced sizes.^20^

**Figure 2:**
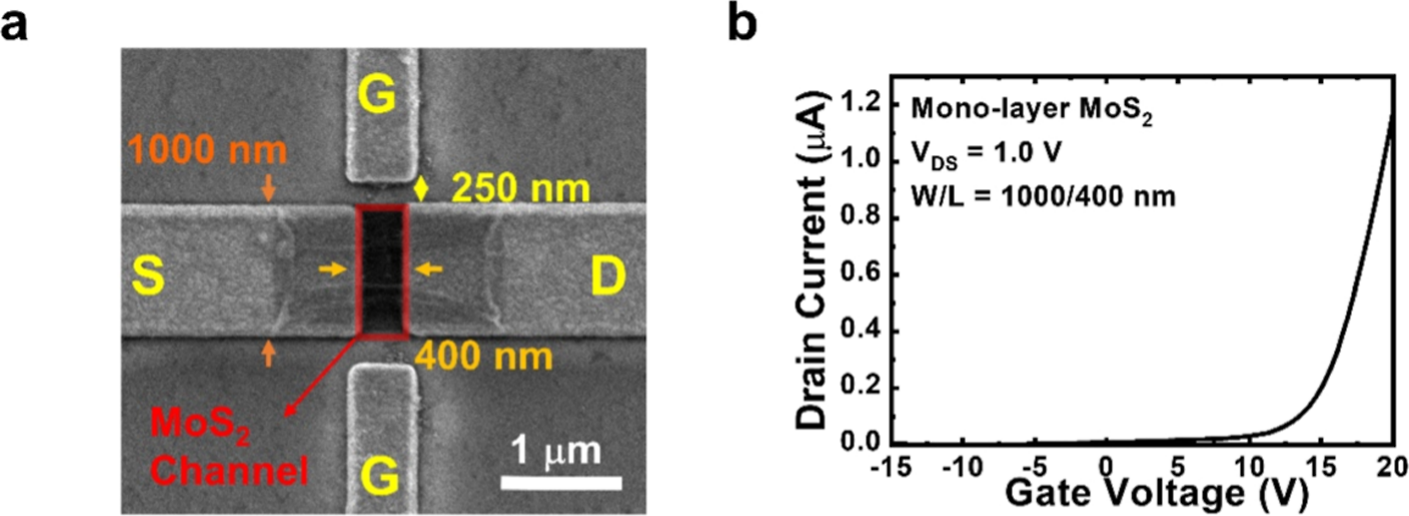
(a) SEM image and (b) transfer curve at *V*_DS_ = 1.0 V of the device with a 1000 nm-wide MoS_2_ channel.

Since the gate electrodes and channel are nearly coplanar, the lateral electrical fields may not modulate the carrier density at the center of the MoS_2_ channel. To investigate the effective channel width of the device, we prepare another device with the same channel length of 400 nm and gate-to-channel separation of 250 nm but a wider channel width of 1000. The SEM image of the device is shown in Figure 2a, and its transfer curve is shown in Figure 2b. Although current modulations under different gate biases are still observable, the ON/OFF ratio of the device decreases to 1 × 10^3^. The lateral gate bias cannot fully deplete the electrons in the 1000 nm wide MoS_2_ channel at a gate bias of −15 V. The transfer curve in the logarithmic scale (Figure S1c in the Supporting Information) would therefore exhibit a high off current for the device. On the other hand, compared with the device with 500 nm wide MoS_2_ channel at a gate bias of 20 V, here the same bias voltage results in a slightly higher drain current around 1.2 μA, indicating that the effective channel width of the device should be around 500 nm. The lateral fields cannot modulate the middle part of the MoS_2_ channel (about 500 nm wide), and hence the MoS_2_ channel is not fully depleted under negative biases. Under positive gate biases, the effective 500 nm channel away from the middle part of MoS_2_ contributes a similar drain current and turn-on behavior to those of the device with a 500 nm wide MoS_2_ channel. In this way, the derived field-effect mobility of the device with a 1000 nm wide MoS_2_ channel would be half of the counterpart of the device with a 500 nm MoS_2_ channel, that is, 30.6 cm^2^·V^–1^·s^–1^. This picture suggests that at the 250 nm gate-to-channel separation, the lateral gate electrodes can modulate the IPGTs well with MoS_2_ channels narrower than 500 nm.

**Figure 3:**
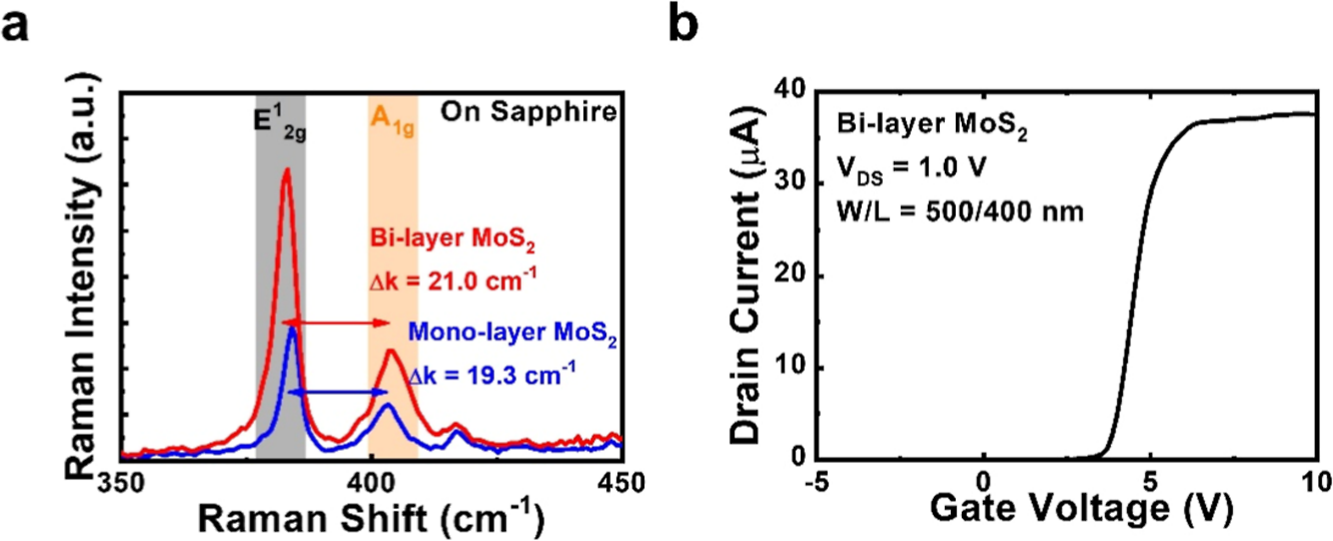
(a) Raman spectra of the sample after one and two times transfers of monolayer MoS_2_. (b) Transfer curve at *V*_DS_ = 1.0 V of the IPGT with bilayer MoS_2_ channel.

It has been demonstrated that a single layer of MoS_2_ can be a protective layer to isolate the negative effect from the substrate to the MoS_2_ channel and therefore enhances the device performance.^20^ To investigate whether the same phenomenon may take place in IPGTs, we first create a film of bilayer MoS_2_ on sapphire substrates through the sequential transferring of monolayer MoS_2_. Compared with the conventional transfer process assisted by poly(methyl methacrylate) (PMMA),^28,29^ the PDMS-stamping method significantly reduces the contaminations remaining on the surfaces of 2D material.^30,31^ Therefore, PDMS transferring is adopted here for the formation of bilayer MoS_2_ film on sapphire substrates. The detailed transferring procedure is shown in the Experiments section. The Raman spectra of the sample after one and two MoS_2_ transfers are shown in Figure 3a. The characteristic Raman peaks E_2g_^1^ and A_1g_ of MoS_2_ can be observed, and the difference Δ*k* between the two Raman peaks increases from 19.3 to 21.0 cm^–1^ with the number of layers, which is typical of monolayer and bilayer MoS_2_ films. The pictures of the sample after one and two MoS_2_ transfers are shown in Figure S3 of the Supporting Information. The complete transfer of monolayer MoS_2_ to either a sapphire substrate (first MoS_2_ layer) or an existing MoS_2_ surface (second MoS_2_ layer) also suggests that the adhesions of sapphire–MoS_2_ and MoS_2_–MoS_2_ interfaces are stronger than that of the MoS_2_–PDMS interface. In this way, we can form bilayer MoS_2_ on sapphire through sequential PDMS stamping without leaving contaminations on the MoS_2_ surfaces. The development of the sequential film transferring technique with wafer-scale and monolayer MoS_2_ provides an easy approach preparing multilayer MoS_2_ films with good layer number controllability across the wafer.

Following the same fabrication process, a bilayer MoS_2_ IPGT with the same channel width/length of 500:400 nm and gate-to-channel separation 250 nm is fabricated. The transfer curve of the device at *V*_DS_ = 1.0 V is shown in Figure 3b. The same curve in the logarithmic scale is shown in Figure S1d of the Supporting Information. We note that the bilayer MoS_2_ IPGT exhibits a drain current level 30–40 times higher than that of the monolayer MoS_2_ IPGT, suggesting the presence of much higher electron density in the former. The lower threshold voltage of the device is consistent with that in the picture. A clear saturation region is also present on the transfer curve of bilayer MoS_2_ IPGT. One possible mechanism responsible for the better performance of the transfer curve may be the efficient electron storage/accumulation in the multilayer MoS_2_ films.^32,33^ From the transfer curve, a quick estimation of field-effect mobility of the bilayer MoS_2_ IPGT reaches 6365.9 cm^2^ V^–1^·s^–1^, and its ON/OFF ratio is over 10^9^ due to the significant drain current level after turn-on. Although such a high mobility may be due to improper usage of the typical formula of field-effect mobility that may need corrections in the case of IPGTs, various device measurements indicate that the architecture of IPGTs can be advantageous for practical applications of electronic devices based on 2D materials. The transfer curves of other five IPGTs with bilayer MoS_2_ channels on the same sample are shown in Figure S4 of the Supporting Information. Their similar device performances have demonstrated the consistency and uniformity of devices, both material-wise and fabrication-wise. The results of the bilayer MoS_2_ IPGT also demonstrate that with further increasing layer numbers in the MoS_2_ channels, even better performances may be obtained for MoS_2_ IPGTs. Further investigation of the influence of MoS_2_ layer numbers to optimized IPGT performances will be conducted in the future.

**Figure 4:**
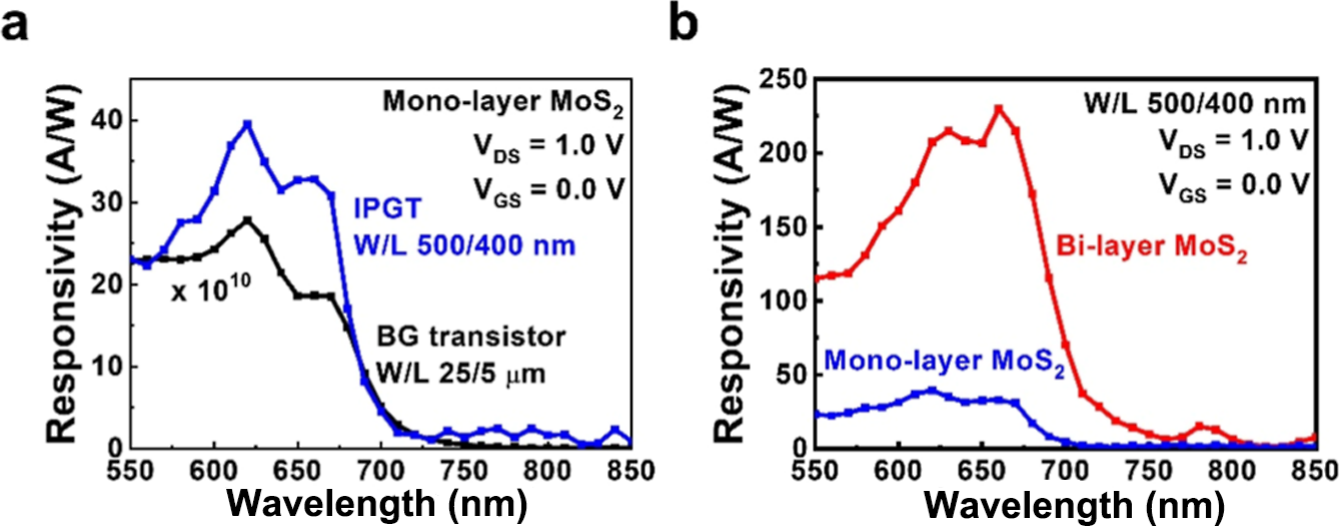
(a) Spectral responsivities at *V*_DS_ = 1.0 V and *V*_GS_ = 0.0 V of the IPGT and BG transistor with monolayer MoS_2_ channels. (b) Spectral responsivities at *V*_DS_ = 1.0 V and *V*_GS_ = 0.0 V of IPGTs with monolayer and bilayer MoS_2_ channels.

Although the small areas of IPGTs in this study are not really suitable for photodetection, the photocurrent measurements of these devices may give more insights on the carrier transport in MoS_2_ channels. For this purpose, we characterize optical spectral responses of IPGTs using a measurement system based on a Xenon (Xe) light source. The schematic diagram of the system setup is shown in Figure S5 of the Support Information. The spectral responsivity of the monolayer MoS_2_ IPGT is shown in Figure 4a. For comparison, the counterpart of a monolayer MoS_2_ BG transistor with a channel width/length of 25:5 μm is also shown in the figure. The *V*_DS_ is set to 1.0 V, and no gate bias is applied to the two devices. As can be told from Figure 4a, the responsivity of the monolayer MoS_2_ IPGT is over 10 orders of magnitude higher than the monolayer MoS_2_ BG transistor. The high responsivity values 30–40 A/W in the wavelength range 600–650 nm also yield a quantum efficiency (QE) of about 80, which reflects the known gain effect of photon conductivity due to the short channels at a fixed applied voltage (*V*_DS_ = 1.0 V). The responsivities at *V*_DS_ = 1.0 V and *V*_GS_ = 0.0 V of IPGTs with monolayer and bilayer MoS_2_ channels are shown in Figure 4b. Although the device geometry is the same for the two devices, another five-time enhancement of responsivity is observed for the bilayer MoS_2_ IPGT. The high responsivity of the device also yields a high QE more than 400 in the wavelength range 600–650 nm, which reflects the more pronounced photogating effect in the bilayer MoS_2_ than in the monolayer MoS_2_.^24^ The same phenomenon of enhanced responsivity values with increasing MoS_2_ layer numbers is also observed in transistors with exfoliated MoS_2_ flakes.^34^ Responsivity values up to 10^4^ A/W are observed with MoS_2_ layer numbers of up to 10. This phenomenon also supports the picture of better electron storage/accumulation of carriers in the bilayer MoS_2_ due to a longer effective recombination time assisted by trap/defect states. With the excess electron storage/accumulation in the bilayer MoS_2_ channel, a more pronounced photoconductive current effectively results in a 5 times higher responsivity.

## Conclusions

In conclusion, we have demonstrated IPGTs with a channel width/length of 500:400 nm and gate-to-channel separation 250 nm made of monolayer and bilayer MoS_2_. The excess electron storage/accumulation in bilayer MoS_2_ significantly enhances the drain current level. In addition to the high drain current, the low OFF current can also be demonstrated through the in-plane gates, which leads to a high ON/OFF ratio over 10^9^. The excess electron storage/accumulation in the short bilayer MoS_2_ channel also plays a part in the high responsivity of 200–230 A/W in the wavelength range 600–650 nm. The IPGT device architecture provides an alternate approach for 2D material transistor fabrications. Without the inferior influence of the dielectric layer on 2D material channels, optimized transistor performances can be achieved by using a dielectric-free device design. The development of sequential film transferring technique with wafer-scale and monolayer MoS_2_ provides an easy approach to preparing layer-number-controllable multilayer MoS_2_ films, which is advantageous for mass production of 2D material devices. The high performance of bilayer MoS_2_ IPGTs has shown the potential for practical applications.

## Experiments

The CVD-grown monolayer MoS_2_ sample used in this work was purchased from 2D Semiconductor Company. The MoS_2_ sample was grown in a 3 in. CVD system. A dual-temperature-zone tube furnace with an 80 mm diameter tube was employed for heating, with a S source and MoO_3_ source, and MoO_3_ itself was heated to 650 °C, while sulfur was heated to 180 °C. The growth process was carried out at a pressure of 8000 Pa for 10 min to grow monolayer MoS_2_ on *c*-plane sapphire substrates. For the transfer of MoS_2_ film, we attach a PDMS substrate to the as-grown MoS_2_/sapphire sample. After peeling off the PDMS/MoS_2_ film from the sapphire substrate in KOH solution, the film is reattached to the other sapphire substrate, and the PDMS film is peeled off from the MoS_2_ surface after high-temperature annealing under atmospheric conditions at 100 °C. This process is to release the strain accumulated in the monolayer MoS_2_ film during the growth. To enhance the adhesion of MoS_2_ to the sapphire surface, the sample is annealed at 150 °C for 2 h under an Ar environment in a hot furnace. Through the sequential transferring of MoS_2_, bilayer MoS_2_ can also be formed on sapphire substrates. For the fabrication of IPGTs, photolithography and metal lift-off are performed to fabricate large electrodes with 100 nm Au deposited at 75 °C. A slightly higher deposition temperature 75 °C than room temperature of the Au electrodes is to decrease the contact resistance at the Au/MoS_2_ interface and not to deteriorate the photoresist for the metal lift-off.^15,25^ The electron-beam lithography is then employed for the deposition of thin extended Au electrodes and definition of channels. The channel is ultimately defined using the RIE with CF_4_ plasma. The spectral responses of the devices are measured using a Horiba iHR 320 monochromator equipped with an optical chopper and a Xe lamp light source. The photocurrents are measured with a Keithley 6487 and a SR830 lock-in preamplifier. The schematic diagram of the measurement system is shown in Figure S5 of the Supporting Information. Raman spectra are obtained using a HORIBA Jobin Yvon HR800UV Raman spectroscopy system with a 532 nm laser. SEM images of the devices are measured using an FEI Inspect F SEM system. Current–voltage characteristics are measured using a Keithley 2636B system with probe stations.

## Supporting Information Available

Transfer curves in logarithmic scale (10^–4^–10^–12^ A) of the four devices with different architectures; SEM image of the monolayer MoS_2_ IPGT with a channel width/length of 500:400 nm and gate-to-channel separation 250 nm; pictures of the sample after one and two-time transfers of monolayer MoS_2_; transfer curves at *V*_DS_ = 1.0 V of five bilayer MoS_2_ IPGTs; and schematic diagram of the measurement system for the spectral responses (PDF)
