# Supplementary material for: Dielectric-Free Molybdenum Disulfide Transistors with In-Plane Gates
Source: ACS Appl Mater Interfaces. 2025 Mar 12;17(12):19020–5. doi: 10.1021/acsami.4c18855 (PMC11955947; doi:10.1021/acsami.4c18855)
Supplement: Supplementary file 1 — am4c18855_si_001.pdf [file am4c18855_si_001.pdf]

Supporting Information

Dielectric-Free Molybdenum Disulfide Transistors with In-plane Gates

Che-Jia Chang[†, ‡], *Shih-Jie Chen*[‡, §], *Tzu-Hsuan Chang*[†], *Po-Tsung Lee*[§], *Shu-Wei Chang*[‡, §], *and Shih-Yen Lin*[*, †, ‡]

[†]Graduate Institute of Electronics Engineering, National Taiwan University, No.1, Sec. 4, Roosevelt Rd., Taipei 10617, Taiwan

[‡]Research Center for Applied Sciences, Academia Sinica, No. 128, Sec. 2, Academia Rd., Taipei 11529, Taiwan

[§]Department of Photonics, National Yang Ming Chiao Tung University, No. 1001, Daxue Rd., East Dist., Hsinchu City 300, Taiwan

*E-mail: shihyen@gate.sinica.edu.tw

The transfer curves corresponding to Figure 1c and 1d, Figure 2b and Figure 3b in logarithmic scale (10^{-4}-10^{-12} A) are shown in Figure S1. As shown in the figure, with a large gate-to-channel separation of 500 nm (Figure S1a), a 10^{-10} A off current is observed for the device at V_{GS} = -40 V. With the gate-to-channel separation reduced to 250 nm (Figure S1b), the off current decreases to 10^{-12} A, which demonstrates that the in-plane gates can more effectively turn off the MoS_2 channel at smaller gate-to-channel separations. With a thick MoS_2 channel up to 1000 nm (Figure S1c), the in-plane gates cannot fully deplete the MoS_2 channel. In this case, a much higher off current of 10^{-9} A is observed for the device. For the device with a bi-layer MoS_2 channel and a 250 nm gate-to-channel separation (Figure S1d), since the bi-layer MoS_2 channel effectively enhances the on current and the in-plane gates can effectively turn off the device, a significantly high ON/OFF ratio up to 10^9 is be observed for the device.

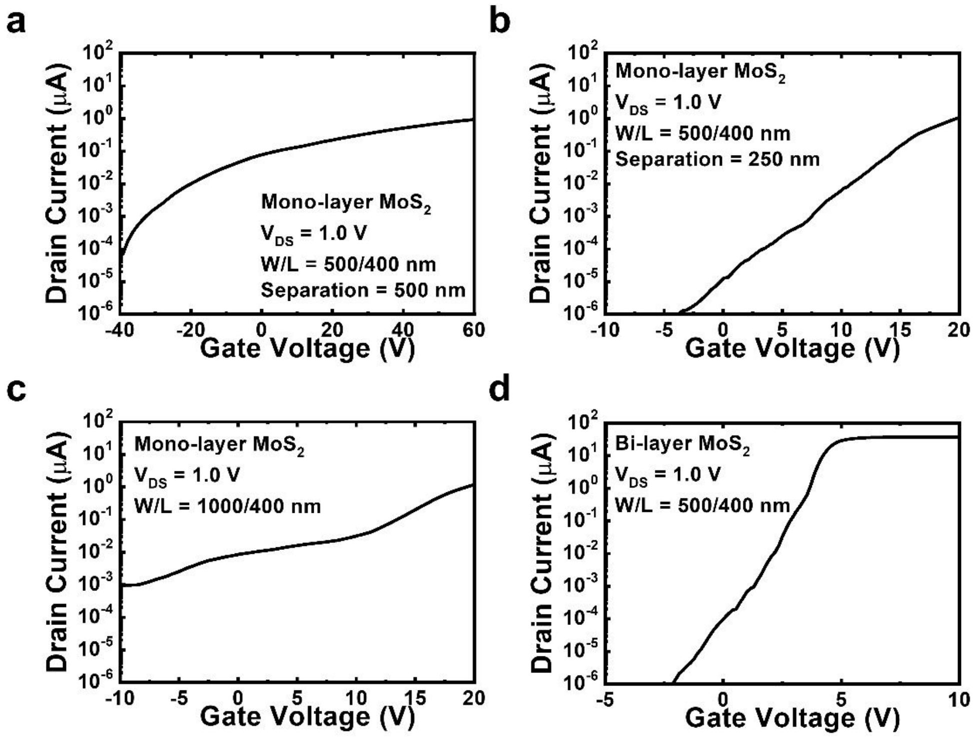

Figure S1. The transfer curves in logarithmic scale (10^{-4}-10^{-12} A) at V_{DS} = 1.0 V of (a) the device with 500 nm gate-to-channel separation and mono-layer MoS_2 channel (W/L 500/400 nm), (b) the device with 250 nm gate-to-channel separation and mono-layer MoS_2 channel (W/L 500/400 nm), (c) the device with 250 nm gate-to-channel separation and mono-layer MoS_2 channel (W/L 1000/400 nm) and (d) the device with 250 nm gate-to-channel separation and bi-layer MoS_2 channel (W/L 500/400 nm).

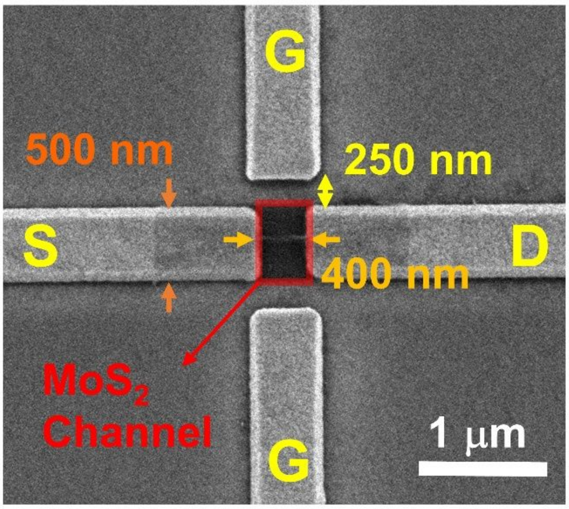

Figure S2. The SEM image of the mono-layer MoS_2 IPGT with channel width/length 500/400 nm and gate-to-channel separation 250 nm.

The pictures of the sample after one- and two- times of mono-layer MoS_2 transferring. Complete MoS_2 film transferring can be observed after each transferring procedure. The picture

taken under an optical microscope of the MoS_2 film edge showing clear blank sapphire, mono-layer to bi-layer MoS_2 distributions.

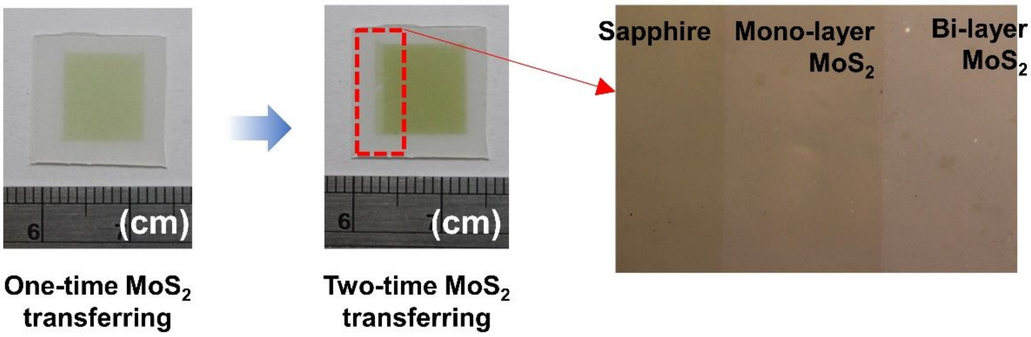

Figure S3. The pictures of the sample after one- and two- mono-layer MoS_2 transferring and the picture taken an optical microscope showing the MoS_2 film edge.

The transfer curves of the other five IPGTs with bi-layer MoS_2 channel on the same sample are shown in Figure S3. Their similar device performances have demonstrated the consistency of IPGTs with the same structure and the uniformity of the sequentially transferred MoS_2.

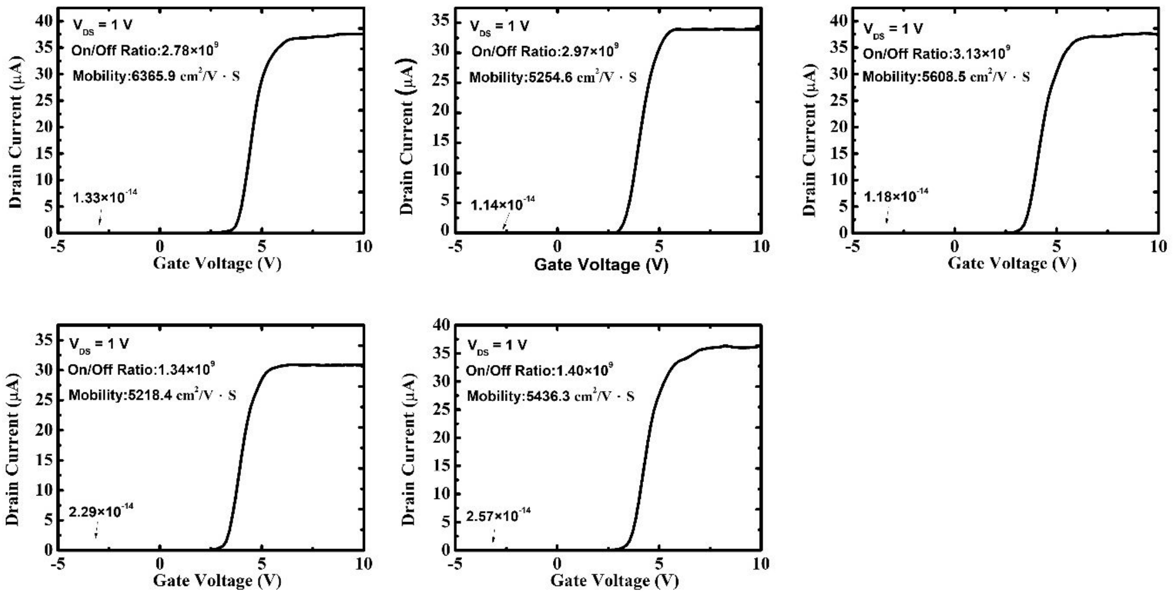

Figure S4. The transfer curves at V_{DS} = 1.0 V of five bi-layer MoS_2 IPGTs.

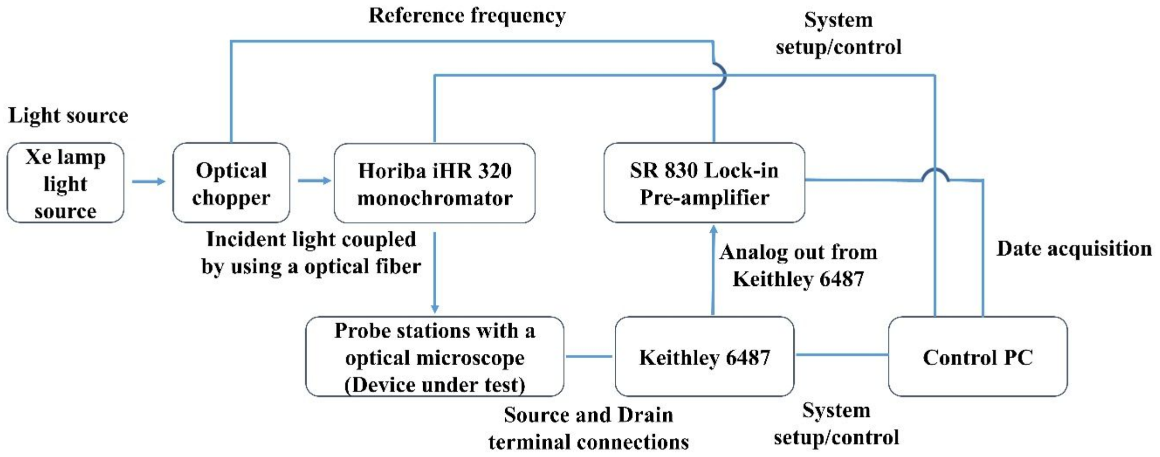

Figure S5. The schematic diagram showing the setup of the spectral response measurement system.
